# Epigenetic Regulation Of Axon Regeneration and Glial Activation in Injury Responses

**DOI:** 10.3389/fgene.2019.00640

**Published:** 2019-07-09

**Authors:** Shalaka Wahane, Dalia Halawani, Xiang Zhou, Hongyan Zou

**Affiliations:** ^1^Nash Family Department of Neuroscience and Friedman Brain Institute, Icahn School of Medicine at Mount Sinai, New York, NY, United States; ^2^Department of Orthopedics, Second Affiliated Hospital of Xi’an Jiaotong University, Xi’an, China; ^3^Department of Neurosurgery, Icahn School of Medicine at Mount Sinai, New York, NY, United States

**Keywords:** neuroepigenetics, axon regeneration, epigenetic regulation, chromatin accessibility, neuroinflammation, spinal cord injury, CNS injury, neural repair

## Abstract

Injury to the nervous system triggers a multicellular response in which epigenetic mechanisms play an important role in regulating cell type-specific transcriptional changes. Here, we summarize recent progress in characterizing neuronal intrinsic and extrinsic chromatin reconfigurations and epigenetic changes triggered by axonal injury that shape neuroplasticity and glial functions. We specifically discuss regeneration-associated transcriptional modules comprised of transcription factors and epigenetic regulators that control axon growth competence. We also review epigenetic regulation of neuroinflammation and astroglial responses that impact neural repair. These advances provide a framework for developing epigenetic strategies to maximize adaptive alterations while minimizing maladaptive stress responses in order to enhance axon regeneration and achieve functional recovery after injury.

## Introduction

Compared to embryonic state or peripheral nervous system (PNS), adult neurons in mammalian central nervous system (CNS) have limited capacity to regenerate after axonal injury. In contrast, axon regeneration and neural circuit rewiring are more effective in non-mammalian nervous systems, which may reflect an evolutionary price paid in exchange for higher complexity in the mammalian CNS. Mounting evidence supports an important role of epigenetics in neurodevelopment and neurodegeneration (Qureshi and Mehler, 2018), however, our understanding of epigenetic regulation of stress responses and adaptive or maladaptive changes in PNS and CNS injury is still in its nascence (Palmisano and Di Giovanni, 2018).

**Figure 1:**
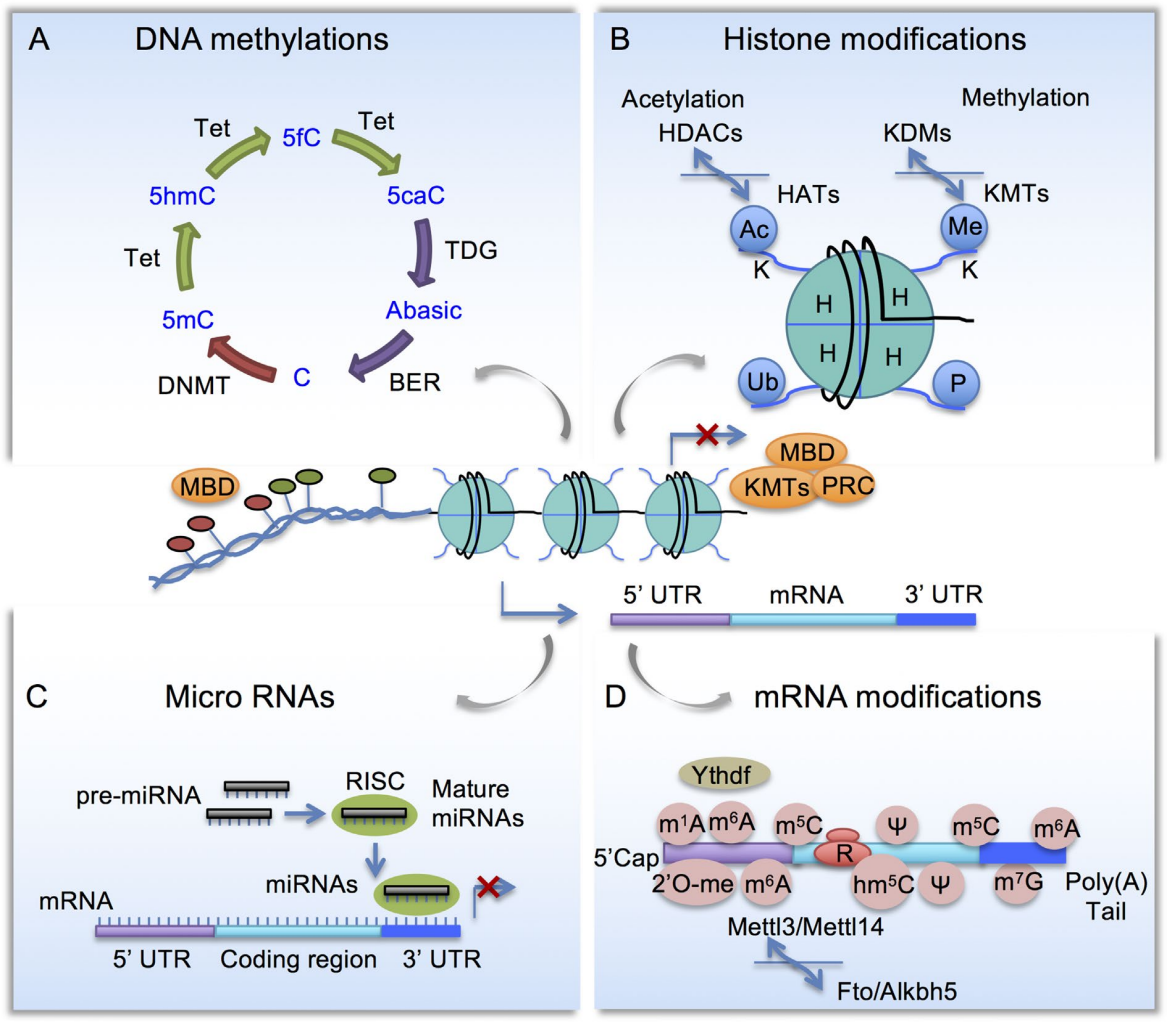
Schematic diagram of epigenetic mechanisms. **(A)** Cytosine methylation and demethylation process. DNMT, DNA methyltransferase; Tet, Ten-eleven translocation methylcytosine dioxygenase; BER, base excision repair enzymes; MBD, Methyl-CpG-binding domain (MBD) proteins. **(B)** Histone modifications. HAT, Histone acetyltransferases; HDAC, histone deacetylases; KMT, lysine methyltransferases; KDM, lysine demethylases; PRC, polycomb repressor complex. **(C)** Micro RNAs are embedded in a multi-protein complex termed RNA-induced silencing complex (RISC) and they repress gene expression by complementary binding to 3’ untranslated region (3’ UTR). **(D)** mRNA modifications include N^6^-methyladenosine (m^6^A), controlled by methyltransferase complex comprised of Mettl3, Mettl14 to install m^6^A, and demethylases Fto and Alkbh5 to remove m^6^A. m^6^A-binding proteins such as YTHDF regulates RNA metabolism.

Since coined by Waddington in the 1940s (Waddington, 1942), epigenetics has evolved in scope to the studies of gene expression changes without altering underlying DNA sequence. Initially referred to as heritable changes of gene expression, epigenetics has since been shown to be highly dynamic, involving writers, readers, and erasers that modify and interpret the epigenetic marks (Kouzarides, 2007). Histone modifications, DNA methylation, and non-coding RNAs are among the best studied, while new modalities continue to emerge. Histones modifications and DNA methylation affect local chromatin structure and protein–DNA interactions, thereby transcriptional output; while non-coding RNAs and modifications of messenger RNA (mRNA) affect RNA metabolism, such as mRNA processing, translation, and decay (**Figure 1**). Epigenetic mechanisms vastly increase the complexity of gene regulation, allowing cells to fine-tune transcriptional responses to the evolving profiles of intrinsic and extrinsic signals in homeostatic and pathological conditions.

Here, we summarize recent progress in understanding neural intrinsic and extrinsic epigenetic mechanisms that govern regenerative capacity of neurons and neuroinflammation and glial response after injury. We define specific epigenetic strategies used to enhance axon regeneration and functional recovery after injury. What emerged from these studies is the epigenetic plasticity in shaping both neuroregenerative and neuroinflammatory pathways, and their diverse roles, with some promoting while others hindering axon regeneration depending on the cell types and neuronal tissues that are most affected. What also comes to light is that pro-regenerative transcription factors (TFs) may require interactions with chromatin regulators to maximize the regenerative effect. Finally, epigenetic modifications also influence the inflammatory responses of microglia and astrocytes, and may be employed for neuroprotection and neural repair. Better understanding of the cell type-specific epigenetic plasticity might provide new avenues to harness its vast potential to promote long-lasting functional recovery after injury.

## The Conditioning Paradigm of Sensory Neurons in Dorsal Root Ganglion

**Figure 2:**
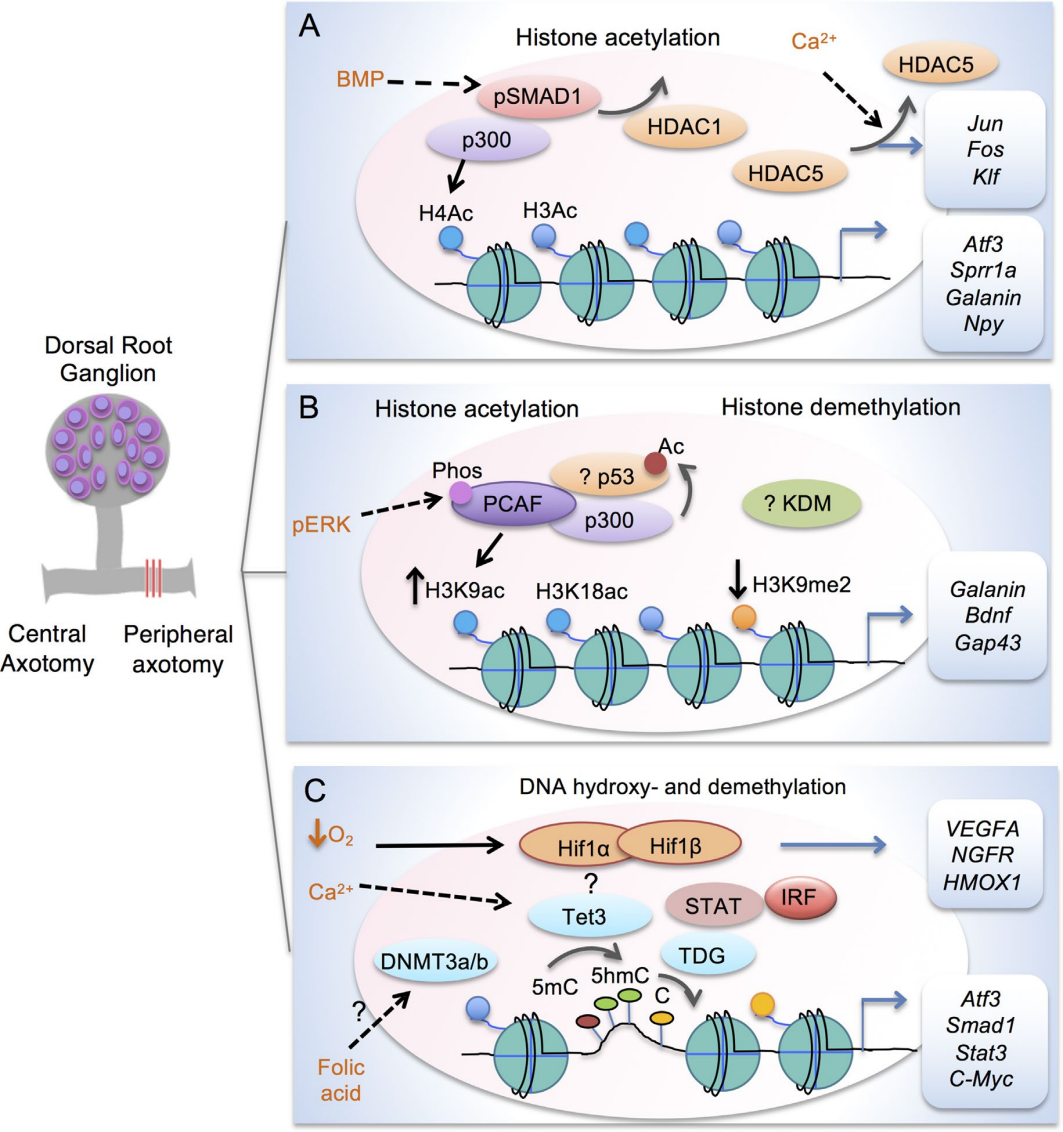
Transcription modules in regulating axon growth potential of DRG neurons after peripheral axotomy. **(A)** Schematic depiction of transcriptional module comprised of Smad1 and p300 to enhance histone acetylation in conditioned DRG at target loci (*Atf3*, *Sprr1a*, *Galanin* and *Npy*). Promoter occupancy of Smad1 helps recruitment of p300 and displacement of HDAC1. Peripheral axotomy also triggers nuclear exit of HDAC5 in response to retropropagation of calcium wave, leading to induction of RAGs (*Jun*, *Fos*, and *Klf*). **(B)** Transcriptional module comprised of p53 and p300/PCAF regulates H3K9ac and H3K18ac at target RAGs (*Galanin*, *Bdnf*, *Gap43* and *Coronin1b*) in conditioned DRG. Acetylation of p53 is also increased by HATs in cortical neurons. Retrograde signaling of pERK results in threonine phosphorylation and nuclear localization of PCAF. In conditioned DRG, H3K9me2 is decreased regulated by a yet unknown KDM. **(C)** Epigenetic factors in mediating DNA hydroxy- and demethylation in conditioned DRG. Peripheral axotomy triggers Tet3 upregulation, which may be dependent on Calcium wave. Tet3 catalyzes conversion of 5mC to 5hmC, while TDG mediates conversion back to C, resulting in RAG induction of *Atf3*, *Smad1*, *Stat3*, and *C-Myc*. Folate may influence DNA methylation through DNMT3a/3b. Tet3 may partner with HIF, STAT, and IRF for 5hmC reconfigurations. Hypoxia stabilizes HIF complex to induce target genes, e.g. *VEGFA*, *NFGR* and* HMOX1*.

After neuronal maturation in the mammalian CNS, it is thought that the pro-axon growth gene program is turned off, and it remains inactivated even after axonal injury. Much effort therefore has been devoted to transcriptome profiling across developmental periods, across species, and PNS vs. CNS, in the hope to identify key pro-growth genes that can promote axon regeneration and functional recovery after CNS injury (reviewed in Blackmore, 2012). Sensory neurons in the dorsal root ganglion (DRG) are unique in that they share features with both PNS and CNS neurons. DRG neurons project a unipolar axon with a peripheral branch that innervates peripheral targets and a central branch that projects into the spinal cord (**Figure 2**). After axotomy, the peripheral, but not the central branch, can initiate robust axon regeneration (McQuarrie and Grafstein, 1973; Richardson and Issa, 1984; Smith and Pate Skene, 1997). Remarkably, peripheral axotomy switches DRG neurons into a growth state that enables not only peripheral but also central branch axon regeneration (Neumann and Woolf, 1999). This so-called conditioning lesion effect is transcription dependent (Smith and Pate Skene, 1997), thus presenting a unique opportunity to study pro-axon growth gene program and the underlying transcriptional mechanisms by comparing gene expression in DRG neurons after peripheral vs. central axotomy.

Earlier efforts have been focused on identifying and characterizing differentially regulated genes or regeneration-associated genes (RAGs) in the conditioning lesion paradigm of DRG through transcriptional profiling (Bonilla et al., 2002; Costigan et al., 2002; Michaelevski et al., 2010; Blesch et al., 2012). However, manipulating individual RAGs, such as ATF3 or STAT3, or simultaneously expressing multiple RAGs turned out insufficient to induce a full regenerative program (Seijffers et al., 2007; Bareyre et al., 2011; Fagoe et al., 2015). Clearly, axon regeneration is a complex process involving multiple RAGs that span diverse functional classes, thus understanding the transcriptional mechanisms, in particular epigenetic regulation, may lead to novel strategies to induce an entire class of RAGs. Indeed, several recent studies revealed that peripheral axotomy of DRG neurons elicits unique chromatin alterations with corresponding transcriptional responses (Cho et al., 2013; Finelli et al., 2013b; Puttagunta et al., 2014). In the following sections, we will review progress made in characterizing the multiple modalities of epigenetic mechanisms involved in regulating RAGs expression and regenerative capacity of conditioned DRG neurons, and when applicable, relate and extend the findings to CNS neurons.

## Histone Modifications in Regulating Axon Growth Gene Program

Histone acetylation is one of the best-studied epigenetic mechanisms that regulate RAGs expression in conditioned DRG (**Tables 1** and **2**). The basic unit of chromatin is known as nucleosome, with DNA wrapping around an octamer of histones (two copies of H2A, H2B, H3 and H4 each). Post-translational modifications of N-terminal histone tails include acetylation, methylation, phosphorylation, ubiquitination, and deimination, etc., among which the best studied are lysine acetylation and lysine methylation (Huang et al., 2014) (**Figure 1B**). Histone acetyltransferases (HATs) add an acetyl group to a specific lysine residue of histone tails and histone deacetylases (HDACs) remove it. In mammalian cells, HAT members include three subgroups: the guanine nucleotide-binding protein subunit alpha transducin (GNAT) family, p300/CREB-binding protein (CBP), and the MYST-family, named after founding members MOZ, Ybf2, Sas2, and TIP60 (Lee and Workman, 2007). The HDAC family has 18 members, grouped into four classes based on structural differences and mechanisms of action (Kouzarides, 2007). In general, histone acetylation facilitates transcriptional activity by neutralizing the positive charge of lysine, leading to open chromatin and increased accessibility by transcriptional regulators, while histone deacetylation leads to chromatin compaction and gene silencing (Mellor, 2005). Histone methylation is catalyzed by lysine methyltransferases (KMTs), and demethylation by lysine demethylases (KDMs). Each member of the KMT (33 known members) and KDM families (22 known members) displays specificity for individual lysine residues as well as mono-, di- and/or trimethylation (Black et al., 2012). Histone tails can also be methylated at arginine residues by protein arginine methyl-transferases (PRMTs) and demethylated by Jumonji domain containing lysine demethylases (reviewed in Tewary et al., 2019). In contrast to histone acetylation, histone methylation can result in either transcriptional activation or repression depending on the identity of the targeted histone residues and the type of methylation. Collectively, these histone modifications (“histone codes”) are recognized by different reader proteins that contain specialized domains such as bromodomains and chromodomains (reviewed in Musselman et al., 2012).

**Table 1:** Histone modifications associated with axon growth potential.

Histone codes	Epigenetic factors	Neuron type	Experimental models	Molecular targets	Ref.
H3ac	HDAC5	Adult DRG neurons	Peripheral nerve regeneration Nuclear exit elicited by retro-propagation of Ca^2+^ wave upon axotomy	Jun, Fos, Klf	(Cho et al., 2013)
H4ac	HDAC1 p300	Adult DRG neurons	Sensory axon regeneration in SCI model Smad1 recruits p300 and displaces HDAC1 at promoters of target genes	Sprr1a, Npy, Galanin, Vip	(Finelli et al., 2013b)
H3K9ac (gene activation) H3K9me2 (gene silencing)	• PCAF • (an H3K9ac-specific HAT) • No changes in H3K18ac, H3K4me2 or H3K27me3 that correlated with RAGs changes	Adult DRG neurons	• PCAF activated by pERK-mediated retrograde signaling after axotomy • AAV-PCAF promotes neurite outgrowth on laminin and myelin and ascending sensory axon regeneration after dorsal column SCI • PCAF^-/-^ DRG neurons show abolished axon growth induced by periphery axotomy in culture and in SCI model	Gap43, Galanin, Bdnf, but not at Sprr1a, Atf3, Hsp27: proximal promoters exhibit enriched H3K9ac and depleted H3K9me2 after peripheral axotomy	(Puttagunta et al., 2014)
H3K9me3	-	Adult DRG neurons	Sciatic nerve injury model UHRF1 interacts with H3K9me3 and DNMTs at the promoter region to repress gene expression by promoter methylation	PTEN, CDKN1A, REST (H3K9me3 enriched at CpG promoter region after peripheral axotomy)	(Oh et al., 2018)
H3K9ac H3K14ac	HDAC (Class I and II) inhibition by TSA, PB increases H3K9ac, H3K14ac CBP/p300 or PCAF knockdown reduces H3K9ac, H3K14ac	P7 Cerebellar granule cells Cortical neurons	• H3K9ac and H3K14ac decline during development • TSA → Increased neurite outgrowth on both permissive and non-permissive substrates (myelin or CSPG) *in vitro*	• Gap43 and Coronin1b (TSA→H3K9ac/ • H3K14ac at promoters) • CBP/p300 and PCAF (TSA→ H3ac at HAT promoters) • p53 (acetylation by TSA)	(Gaub et al., 2010)
H3K18ac	p300	RGC (retinal ganglion cell)	• H3K18ac and p300 levels decline during RGC maturation • Adenoviral overexpression of p300→ axon regeneration in optic N crush model • TSA→ no RGC regeneration, but enhances RGC survival	• p300 binds to promoters of Gap43, Coronin1b and Sprr1a • p300 increases H3K18ac • p300 increases p53 ac and C/EBP ac	(Gaub et al., 2011)
H3K4me3 (euchromatin marker) H3K27me3 (hetero-chromatin marker)	-	Embryonic vs. postnatal cortical neurons	Cortices from E15, P3, P7, P14, P21 and adult In postnatal cortical neurons, overexpression of KAT2A and KAT2B (PCAF) did not affect neurite outgrowth, nor did HDAC inhibitor TSA and Scriptaid	• H3K4me3 enriched at promoters of Sprr1a, Integrin α7, Galanin, and Gap43 in E15 vs. adult cortex, but not at Hsp27 and Cap23 • H3K27me3 enriched in adult cortex vs. embryonic in these RAGs	(Venkatesh et al., 2016)

**Table 2:** Epigenetic regulation of RAG expression.

RAG	Epigenetic modification	Localization	Neuron type	Epigenetic modifier	Gene regulation	Reference
Atf3	H4ac	Promoter	Adult DRG SNL 6 hr	HDACi (MS275) no effect	Gene upregulation correlated with H4ac enrichment at promoter 6 hr after SNL No H4ac enrichment or gene induction with HDAC1 inhibitor MS275 In N2A cells, pSmad1 promoter binding recruits p300	(Finelli et al., 2013b)
	DNA demethylation	Distal Enhancers, but not promoter show DNA demethylation after SNL	Adult DRG neurons SNL day 1	Tet3 TDG	Tet3 binds to Distal Enhancer Tet3 KD or TDG KD attenuates Atf3 induction in conditioned DRG DNA demethylation at DE1 of Atf3 after SNL requires Tet3	(Weng et al., 2017)
	5hmC	Gene body	Adult DRG SNL day 1	Tet3	Gene induction correlates with 5hmC gain	(Loh et al., 2017)
	m^6^A tagging	mRNA	Adult DRG SNL day 1	Mettl14 Ythdf1	Increase in both m^6^A-tagged and total Atf3 mRNA levels after SNL, Gained new m^6^A sites upon SNL Loss of m^6^A tagging of Aft3 mRNA reduces ATF3 protein translation	(Weng et al., 2018)
Bdnf	H3K9ac gain H3K9me2 loss	Promoter	Adult DRG SNL day 1	PCAF	Gene induction PCAF promoter occupancy increases upon SNL	(Puttagunta et al., 2014)
	5hmC	Gene body (last exon)	Adult DRG SNL day 1	Tet3	Gene induction correlates with 5hmC gain	(Loh et al., 2017)
Myc	DNA demethylation	Distal enhancer sites show DNA demethylation after SNL	Adult DRG neurons SNL day 1	Tet3 TDG	Tet3 KD or TDG KD attenuates Myc induction in conditioned DRG	(Weng et al., 2017)
Coronin 1b	H3ac	–	Cerebellar granule cells	HDACi (TSA)	TSA induces Coronin1b expression	(Gaub et al., 2010)
	H3ac	Promoter	RGC Optic Nerve crush	p300	Upregulated with AV-p300 p300 promoter occupancy	(Gaub et al., 2011)
Gadd45a	DNA methylation	Promoter	Rat SCI with SNL	Dnmt3a Dnmt3b	Upregulation Bilateral dorsal column transection	(Iskandar et al., 2010)
	m^6^A tagging	mRNA	Adult DRG SNL day 1	–	Increase in both m^6^A-tagged and total mRNA levels after SNL	(Weng et al., 2018)
Galanin	H4ac	Promoter	Adult DRG SNL	HDACi (MS275)	Gene upregulation correlated with H4ac enrichment at promoter 6 hr after SNL HDAC1 inhibitor leads to H4ac enrichment and gene induction In N2A cells, pSmad1 promoter binding displaces HDAC1 binding	(Finelli et al., 2013b)
	H3K9ac gain H3K9me2 loss	Promoter	Adult DRG SNL day 1	PCAF	Gene induction PCAF promoter occupancy increases upon SNL	(Puttagunta et al., 2014)
	H3K4me3	Promoter	Developing cortex	–	Enriched in E15 cortex and gradual decline during maturation	(Venkatesh et al., 2016)
	H3K27me3	Promoter	Developing cortex	–	Low in E15 cortex, gradual increase during maturation	(Venkatesh et al., 2016)
Gap43	H3ac	–	Cerebellar granule cells	HDACi (TSA)	TSA induces Gap43 expression	(Gaub et al., 2010)
	H3ac	Promoter	RGC Optic nerve crush	p300	Upregulation with AV-p300 p300 promoter occupancy	(Gaub et al., 2011)
	H3K9ac gain H3K9me2 loss	Promoter	Adult DRG SNL day 1	PCAF	Gene induction PCAF promoter occupancy increase upon SNL	(Puttagunta et al., 2014)
	H4ac	Promoter	Adult DRG	HDACi (MS275) no effect	No H4ac enrichment at promoter 6 hr after SNL No H4ac enrichment or gene induction with HDAC1 inhibitor	(Finelli et al., 2013b)
	H3K4me3	Promoter	Developing cortex	–	Enriched in E15 cortices, gradual decline during maturation	(Venkatesh et al., 2016)
	H3K27me3	Promoter	Developing cortex	–	Low in E15 cortices, gradual increase during maturation	(Venkatesh et al., 2016)
	No change in DNA demethylation	Distal enhancer	Adult DRG SNL	TET3/TDG not involved	No change in DNA methylation status by bisulfite sequencing	(Weng et al., 2017)
Integrin α7	H3K4me3	Promoter	Developing cortex	–	Enriched in E15 cortex, gradual decline during maturation	(Venkatesh et al., 2016)
	H3K27me3	Promoter	Developing cortex	–	Low in E15 cortex, gradual increase during maturation	(Venkatesh et al., 2016)
Jun	m^6^A tagging	mRNA	Adult DRG SNL day 1	–	Increase in both m^6^A-tagged and total mRNA levels of Jun after SNL	(Weng et al., 2018)
Npy	H4ac	Promoter	Adult DRG SNL	HDACi (MS275)	Gene upregulation correlated with H4ac enrichment at promoter after SNL HDAC1 inhibitor MS275 results in H4ac enrichment and gene induction In N2A cells, pSmad1 promoter binding displaces HDAC1	(Finelli et al., 2013b)
PTEN	DNA methylation H3K9me3	CpG promoter region	Adult DRG SNL	DNMTs UHRF1	H3K9me3 enriched at the CpG promoter region of PTEN after SNL, UHRF1 interacts with DNMTs to represses PTEN via promoter methylation	(Oh et al., 2018)
REST	microRNA DNA methylation H3K9me3	3’UTR CpG promoter region	Adult DRG SNL	miR-9 DNMTs UHRF1	Upon SNL, transient increase of REST via downregulation of miR-9, later, reduced REST transcription via UHRF1-mediated promoter methylation	(Oh et al., 2018)
SIRT1	microRNA	microRNA	Adult DRG neurons	miR-138	SIRT1 induced after SNL, required for peripheral nerve regeneration, Downregulate GSK3β, Smad1 acts downstream of miR-26a-GSK3β pathway PTEN not affected	(Jiang et al., 2015)
	–	–	Embryonic cortical neurons	–	Promote neurite outgrowth and cell survival through mTOR signaling	(Guo et al., 2011b)
Smad1	H4ac	Promoter	Adult DRG SNL	HDACi (MS275) no effect	Gene upregulation correlated with H4ac enrichment at promoter 6 hr after SNL No H4ac enrichment or gene induction with HDAC1 inhibitor	(Finelli et al., 2013b)
	5hmC loss	Introns	Adult DRG SNL day 1	Tet3	Two introns both showing 5hmC loss after SNL	(Loh et al., 2017)
	DNA demethylation	–	Adult DRG SNL day 1	Tet3 TDG	Tet3 KD or TDG KD attenuates Smad1 induction in conditioned DRG	(Weng et al., 2017)
Sox11	m^6^A tagging	mRNA	Adult DRG SNL day 1	–	Increase in both m^6^A-tagged and total mRNA levels of Sox11 after SNL	(Weng et al., 2018)
Sprr1a	H4ac	Promoter	Adult DRG SNL day 1	HDACi (MS275)	Gene upregulation correlated with H4ac enrichment at promoter 6 hr after SNL HDAC1 inhibitor leads to H4ac enrichment and gene induction In N2A cells, pSmad1 promoter binding displaces HDAC1 and recruits p300	(Finelli et al., 2013b)
	H3K9ac loss	Promoter	Adult DRG SNL	–	Gene induction, but H3K9ac loss	(Puttagunta et al., 2014)
	H3ac	Promoter	RGC Optic nerve crush	p300	Upregulated with AV-p300 p300 promoter occupancy	(Gaub et al., 2011)
	H3K4me3	Promoter	Developing cortex	–	Enriched in E15 cortex, gradual decline during maturation	(Venkatesh et al., 2016)
	H3K27me3	Promoter	Developing cortex	–	Low in E15 cortex, gradual increase during maturation	(Venkatesh et al., 2016)
STAT3	DNA demethylation	–	Adult DRG SNL day 1	Tet3 TDG	Tet3 KD or TDG KD attenuates STAT3 induction in conditioned DRG	(Weng et al., 2017)
Tet3	m^6^A tagging	mRNA	Adult DRG SNL	–	Increase in m^6^A-tagged Tet3 mRNA after SNL, gained new m^6^A sites upon SNL	(Weng et al., 2018)
UHRF1	microRNA	3’UTR	Adult DRG SNL	miR-9	Gene induction after SNL, target of miR-9	(Oh et al., 2018)
Vip	H4ac	Promoter	Adult DRG SNL	HDACi (MS275)	Gene induction HDAC1 inhibitor leads to H4ac enrichment and gene induction In N2A cells, pSmad1 promoter binding displaces HDAC1	(Finelli et al., 2013b)

AV, adenovirus; HDACi: HDAC inhibitor; KD, knockdown; SNL, sciatic nerve lesion.

In the conditional lesion paradigm, it was found that peripheral but not central axotomy results in global enrichment of histone H3 and H4 acetylation (H3ac and H4ac) in DRG neurons (Finelli et al., 2013b; Puttagunta et al., 2014) (**Figure 2A** and **Table 1**). Functionally, elevating histone acetylation levels by HDAC inhibitors (HDACi) can induce multiple RAGs in DRG neurons. For instance, administrating MS275, a selective inhibitor of HDAC1, can orchestrate transcriptional change of ∼50% of RAGs examined, and significantly enhance axon growth potential and sensory axon regeneration in a dorsal column spinal cord injury (SCI) model (Finelli et al., 2013b). Moreover, *in vitro*, axotomy of DRG neurons triggers a retro-propagation of a calcium wave to the soma, which elicits nuclear export of HDAC5, leading to elevated H3 acetylation levels and RAGs induction (Cho et al., 2013).

The next question is which lysine residue modifications and which HAT/HDAC are specifically involved in transcriptional regulation of RAGs. Puttagunta et al. (2014) performed a comprehensive screen by chromatin immunoprecipitation (ChIP) in conditioned DRG to define lysine residue-specific histone modifications at proximal promoters of RAGs, including histone modifications known to correlate with active gene transcription (H3K9ac, H3K18ac, H3K4me2) or with gene silencing (H3K9me2, and H3K27me3). Only H3K9ac and H3K9me2 showed peripheral axotomy-triggered differential changes with consistent transcriptional changes of known RAGs, with H3K9ac enriched and H3K9me2 depleted at the promoter of *Galanin*, *Bdnf* and *Gap43* (**Figure 2B** and **Table 1**). Consistently, ChIP analyses showed that PCAF, also known as K (lysine) acetyltransferase 2B (KAT2B), an H3K9ac-specific acetyltransferase, was enriched at these promoters. Overexpression of PCAF promoted neurite outgrowth from DRG neurons on both laminin and myelin substrates and enhanced ascending sensory axon regeneration in the dorsal column lesion SCI model. Additionally, retrograde transport of pERK after peripheral axotomy was required for PCAF phosphorylation and nuclear localization, thus providing a mechanism of how injury signals from axotomy are linked to nuclear events and chromatin modifications.

A recent study unveiled the involvement of CBP-mediated histone acetylation in the epigenetic reprogramming in proprioceptive DRG neurons triggered by enhanced neuronal activity through environmental enrichment, and this results in increased regenerative potential through RAGs induction (Hutson et al., 2019). Pharmacological activation of CBP/p300 enhances sprouting of both descending motor and ascending sensory axons leading to functional recovery in both mouse and rat SCI models (Hutson et al., 2019).

An important question is whether epigenetic mechanisms that are effective at promoting peripheral axon regeneration of DRG neurons may also work in CNS neurons. In the CNS, efforts to modulate chromatin acetylation status resulted in mixed findings (**Table 1**). For instance, Trichostatin A (TSA), a broad HDACi, enhanced neurite outgrowth in cultured neonatal cerebellar granule cells (CGNs) (Gaub et al., 2010), but not in retinal ganglion cells (RGCs) (Gaub et al., 2011). Similarly, HDACi alone failed to promote axon growth in postnatal cortical neurons (Venkatesh et al., 2016). These divergent findings indicate that the efficacy of HDACi may depend on the dynamic state of HATs and HDACs in neuronal subtypes. Indeed, DRGs demonstrate basal expression levels of p300 (Finelli et al., 2013b; Usoskin et al., 2015). In RGCs, p300 is developmentally regulated and its expression remained repressed after optic nerve injury (Gaub et al., 2011). Activation of the regeneration program following optic nerve injury requires p300 expression, which increases acetylation of both histone and non-histone target genes (i.e., H3K18, p53, and C/EBP) and RAGs induction (Gap43, Coronin1b, and Sprr1a) (Gaub et al., 2011). Likewise, in cortical neurons and CGNs, TSA can increase the expression of CBP/p300 and PCAF *via* H3K9ac and H3K14ac enrichment, which in turn mediate acetylation of histones and p53 to induce p53 target genes (Gap43 and Coronin1b) (Gaub et al., 2010). In cortical neurons and facial motoneurons, CBP/p300 works with p53 at promoters of RAGs such as Gap43 (Di Giovanni et al., 2006; Tedeschi et al., 2009). As summarized in **Table 2**, different RAGs display distinct histone modification patterns and engage different epigenetic modifiers in different neurons. For instance, promoters of *Sprr1a* and *Atf3* showed elevated H4ac but not H3K9ac enrichment in conditioned DRG (Finelli et al., 2013b; Puttagunta et al., 2014).

One caveat of using HDAC inhibitors is that HDACs have non-histone substrates, so their inhibition may affect non-transcriptional pathways that are linked to axon growth. For instance, HDAC5, along with HDAC6 and SIRT2, also have cytoplasmic functions in deacetylating tubulins and microtubules and regulating neurite outgrowth (Cho and Cavalli, 2014). SIRT1 supports neurite outgrowth in the developing cortical neurons, reportedly through inhibiting mTOR signaling (Guo et al., 2011b). In summary, these findings stress the need to fine-tune epigenetic strategies for targeted chromatin remodeling to account for intrinsic cell-type differences in chromatin state and pro-regenerative gene programs.

## DNA Methylation and Hydroxymethylation in Regulating Axon Regeneration Capacity

DNA methylation status, also known as methylome, refers to cytosine methylation (5mC) that occurs primarily on CpG dinucleotides (**Figure 1A**). CpG islands (CGI) are genomic areas of high CpG density, typically near gene promoters or enhancers. Generally, CGI are hypomethylated for actively transcribed genes, but heavily methylated for silenced genes. Methyl-CpG-binding domain (MBD) proteins preferentially recognize methylated DNA, serving as readers of the methylome (Klose and Bird, 2006). DNA methyltransferases (DNMTs) use S-adenosyl-L-methionine as methyl group donor to convert C to 5mC, with DNMT1 involved in maintenance of DNA methylation patterns during DNA replication (Smith et al., 1992), and DNMT3a and DNMT3b in *de novo* DNA methylation (Okano et al., 1999). Cytosine demethylation, on the other hand, occurs through an intermediate base, 5-hydroxymethylcytosine (5hmC), catalyzed by the Ten-eleven translocation (Tet) methylcytosine dioxygenase family (Tet1-3) (Wu and Zhang, 2010). Tet enzymes can iteratively oxidize 5hmC to 5-formylcytosine (5fC) and 5-carboxycytosine (5caC), which can then be removed by thymine DNA glycosylase (TDG)-mediated base excision repair pathway through Activation-induced cytidine deaminase (AID) or Apolipoprotein B mRNA editing enzyme catalytic (APOBEC) family proteins (Guo et al., 2011a; Hackett et al., 2013). 5hmC modifications are much more abundant in the CNS than in embryonic stem (ES) cells (Tahiliani et al., 2009; Song et al., 2011), and may have regulatory roles in its own right (Hahn et al., 2013). 5hmC is enriched in gene bodies that are highly expressed, suggesting a potential role for 5hmC in activating and/or maintaining gene expression (Song et al., 2011).

Earlier studies demonstrated that folate can regulate axon regeneration through DNA methylation (Iskandar et al., 2010). In our screen for differentially regulated epigenetic factors in adult DRG after peripheral axotomy, we identified Tet3 as specifically upregulated in conditioned DRG, along with elevated 5hmC levels (Loh et al., 2017). We then conducted a genome-wide survey of 5hmC distribution and dynamics in adult DRG under three different regenerative states: growth state after peripheral axotomy, naïve state with no injury, and a state refractory to axon regeneration after central axotomy (Loh et al., 2017). Genomic analyses revealed that a majority of differentially 5-hydroxymethylated regions (DhMRs) (∼55%) in DRG occur in gene bodies and ∼30% in intergenic regions; moreover, ∼90% of DhMRs were in “open sea” and less than 0.5% on CGIs. The relative importance of DNA hydroxy- or demethylation in CGIs vs. gene bodies in RAGs regulation and axon regeneration of DRG neurons vs. RGCs remains to be elucidated. New tools, such as APOBEC-coupled epigenetic sequencing (ACE-seq), a bisulfite-free method, now allows mapping of 5hmC at single-base resolution with low DNA input and without degrading DNA (Schutsky et al., 2018). Functional annotation of DhMR-associated genes showed that the most divergent functional categories were related to axon growth, neurites outgrowth, and neurite regeneration (Loh et al., 2017), and the top DhMR-associated pathways included phosphatase and tensin homolog (PTEN) and BMP signaling, both important for axon growth and regulation (Liu et al., 2010; Parikh et al., 2011; Finelli et al., 2013a). The epigenomic maps of 5hmC distribution revealed that conditioning lesion resulted in unique acquisition or loss of 5hmC modifications in about half of all RAGs. Unexpectedly, even though central lesion does not upregulate Tet3 expression, it also resulted in widespread 5hmC reconfigurations, but with little overlap with those triggered by peripheral axotomy. This suggests that the central lesion engages distinct 5hmC modifications, which may constitute a roadblock for regeneration. It is worth mentioning that earlier DNA methylation studies have been heavily focused on CGI at gene promoters, which may miss changes occurring elsewhere. Indeed, when DNA methylation microarrays were used to study promoter and CGI DNA methylation in conditioned DRG, only a modest number of genes, and none of the RAGs examined, displayed differential methylation between peripheral vs. central axotomy (Puttagunta et al., 2014).

At the same time, Weng et al. (2017) also discovered induction of Tet3 and elevated 5hmC levels in conditioned adult DRG neurons. Tet3 induction after peripheral axotomy may be dependent on a retrograde Ca^2+^ wave. Tet3 is required for 5hmC enrichment and induction of RAGs such as Atf3, Smad1, STAT3, and c-Myc, but not Gap43; and it binds to distal enhancer region of Atf3 and c-Myc (**Figure 2C** and **Table 2**). TDG knockdown attenuated induction of a similar set of RAGs, suggesting that complete DNA demethylation, not 5hmC increase alone, mediates RAGs induction to unlock axon growth potential of adult DRG neurons. Interestingly, while Tet3 is required for functional peripheral axon regeneration of DRG neurons, Tet1 is required for PTEN-deletion-induced optic nerve regeneration of RGCs (Weng et al., 2017), indicating that RAGs expression may require cell-type specific DNA demethylation gene pathways.

Besides regulating DNA demethylation, Tet3 can also recruit O-linked β-N-acetylglucosamine transferase (OGT) to chromatin (Ito et al., 2014), and OGT catalyzes the addition of N-acetylglucosamine to serine/threonine residues of many proteins, including histones; OGT deletion in DRG neurons results in decreased axonal growth (Su and Schwarz, 2017).

A recent study unveiled that DNA methylation, which generally leads to gene silencing, also contributes to regenerative responses of conditioned DRG neurons (Oh et al., 2018). Treatment with RG108, a direct DNMT inhibitor, reduces peripheral nerve regeneration in a sciatic nerve injury model. Periphery axotomy induces ubiquitin-like containing PHD ring finger 1 (UHRF1) and a transient increase of transcriptional regulator RE1 silencing transcription factor (REST), both downstream targets of miR-9. UHRF1 recognizes methyl groups on histone H3, specifically H3K9me3, and recruits DNMTs to promoter region to silence gene expression of PTEN and restrict REST expression *via* promoter methylation, leading to enhanced peripheral axon regeneration (Oh et al., 2018). REST upregulation in DRG also contributes to the transition from acute to chronic pain after peripheral nerve injury through repressed expression of Chrm2, a muscarine cholinergic receptor (Zhang et al., 2018a). Together, these studies demonstrate the complex role of DNA (de)methylation pathways in blocking or promoting axon regeneration, depending on target genes. The dynamics of DNA methylation and hydroxymethylation and their roles in CNS axon regeneration are poorly understood. The challenge is to define loci-specific changes in 5C, 5mC and 5hmC patterns and their functions in governing axon regenerative capacity in specific subtypes of CNS neurons. It remains to be seen whether the identification of critical genomic loci may enable the utilization of Cas9-directed DNA methylation editing to enhance axon regeneration in a cell-specific manner (Liu et al., 2016).

## microRNAs in Regulating Intrinsic Axon Growth Potential

MicroRNAs (miRNAs) are a class of small (∼22 nucleotides) noncoding RNAs that regulate post-transcriptional gene expression (Bartel, 2004). miRNAs are embedded in a multi-protein complex termed RNA-induced silencing complex (RISC) and bind in a complementary fashion to the 3’ untranslated region (3’ UTR) of mRNAs and suppress protein expression by preventing translation and/or promoting mRNA degradation (**Figure 1C**). Each individual miRNA has multiple targets, thus capable of concurrently modulating expression of multiple genes. Each cell type in the CNS has a distinct miRNA profile that contributes to the specification and maintenance of distinct neuronal and glial phenotypes (Jovičić et al., 2013).

In animals with deletion of Dicer, which is critical for microRNA processing, sensory neurons show impaired axon regeneration (Wu et al., 2012). Gene profiling revealed differential expression of many microRNAs in adult DRG neurons after peripheral axotomy (Strickland et al., 2011; Zhang et al., 2011; Zhou et al., 2011). For instance, miR-138 controls axon growth of adult DRG neurons through a mutual negative feedback loop with SIRT1, an NAD-dependent HDAC (Liu et al., 2013) (**Table 3**). In naïve DRG neurons, baseline level of miR-138 is high to inhibit axon growth through suppression of SIRT1 expression. Peripheral axotomy leads to SIRT1 upregulation, which represses miR-138 transcription; this in turn results in sustained elevation of SIRT1 and enhanced axon regeneration in a sciatic nerve injury model (Liu et al., 2013). In another study, miR-26a was found to be required for axon regeneration of adult DRG sensory neurons *in vitro* and in a sciatic nerve injury model (Jiang et al., 2015) (**Table 3**). MiR-26a targets specifically glycogen synthase kinase 3β (GSK3β), and Smad1 acts downstream of miR-26a-GSK3β pathway to control sensory axon regeneration.

**Table 3:** miRNA in regulating axon growth and regeneration.

miRNA	Neurons	Function	Molecular targets	Reference
miR-135a miR-135b	Neonatal hippocampal neurons RGCs	Promote axon growth and axon branching of hippocampal neuron Facilitates RGC axon regeneration in optic nerve injury model	KLF4	(van Battum et al., 2018)
miR-9	Embryonic cortical neurons	Inhibit axonal extension	Map1b (cytoskeletal protein) repressed locally in axon	(Dajas-Bailador et al., 2012)
	Adult DRG neurons	miR-9 downregulation after sciatic nerve injury is critical for axon regeneration	UHTF1, REST (for epigenetic silencing of PTEN through DNA methylation)	(Jiang et al., 2017), (Oh et al., 2018)
miR-124	Embryonic hippocampal neurons	Promote axon branching and maturation	RhoG (small GTPase)	(Franke et al., 2012)
miR-138	Conditioned adult DRG neurons	Inhibit axon growth in sciatic nerve injury model	SIRT1 (class III HDAC)	(Liu et al., 2013)
miR-26a	Adult DRG neurons	Required for peripheral axon regeneration in sciatic nerve injury model	GSK3β, Smad1 acts downstream of miR-26a-GSK3β pathway PTEN not affected	(Jiang et al., 2015)
	Neonatal rat cortical neuron	Support neurite outgrowth	Suppress PTEN	(Li and Sun, 2013)
miR-17-92	Embryonic rat cortical neuron	Expressed in distal axons, Enhance axon growth	Suppress PTEN at distal axons	(Zhang et al., 2013)
miR-222	Adult DRG neurons	Differentially regulated after sciatic nerve injury, Promote axon growth	Suppress PTEN	(Zhou et al., 2012)

Recent studies reveal a large number of microRNAs with regulatory roles for axonal outgrowth, including miR-9, 17-92, 21, 26a, 30b, 133b, 135a, 135b, 138, 210, 222, and 431 (**Table 3**, also see review in Weng et al., 2016). For instance, in embryonic cortical neurons, miR-9 inhibits axon extension while augmenting axon branching through translational repression of microtubule-associated protein 1b (Map1b) locally in axon (Dajas-Bailador et al., 2012). In adult DRG neurons, peripheral axotomy results in downregulation of miR-9, which is critical for axon regeneration (Jiang et al., 2017). As mentioned above, REST and UHRF1 are both downstream targets of miR-9, and UHRF1 enhances regenerative capacity of DRG neurons through gene silencing of PTEN and REST *via* promoter methylation, thus providing an example of interplay between different epigenetic mechanisms (Oh et al., 2018). In embryonic hippocampal neurons, CNS-specific miR-124 promotes axonal branching through suppressing the small GTPase RhoG (Franke et al., 2012). Van Battum et al. recently performed an miRNome-wide functional screen of >1000 miRNAs in a neuronal cell line, SH-SY5Y cells, and identified miR-135a and miR-135b as promoters of axon growth. The promoting effect of these microRNAs were confirmed in primary hippocampal neurons. Intravitreal injection of miR-135s facilitated RGC axon regeneration after optic nerve injury. KLF4, an inhibitor of axon regeneration, was identified as a target of miR-135 (van Battum et al., 2018).

To relate the findings in different neurons is challenging as different microRNAs exert different functions and affect different target genes in a cell-type specific manner, with some promoting while others inhibiting axon growth. One example is PTEN: while PTEN reportedly was not affected by miR-26a in adult DRG neurons; but in neonatal rat cortical neurons, miR-26a supported neurite outgrowth by repressing PTEN expression (Li and Sun, 2013) (**Table 3**). Interestingly, in adult sensory neurons, miR-222 is shown to target PTEN and promotes axon growth (Zhou et al., 2012), while in embryonic rat cortical neurons, the miR-17-92 cluster enhances axon growth through targeting PTEN (Zhang et al., 2013). Clearly, PTEN is regulated by different miRNAs in a cell-type specific manner. Furthermore, miRs are under the regulatory control of long noncoding RNA (LncRNA), which can function to decoy or “sponge” miRs to limit their function *in vivo* (Ebert and Sharp, 2010). Future characterization of LncRNA-miRNA-mRNA networks in regenerating neurons may provide new avenues to enhance axon growth potential in injured tissue.

## Epitranscriptomic Regulation of Axon Regeneration

In analogy to DNA modifications, various covalent modifications are also present on RNA transcripts (**Figure 1D**). Epitranscriptomic modifications of RNA can influence stability, translation, and non-coding RNA function, thus adding an additional layer of complexity to gene regulation (Zhao et al., 2017). RNA modifications include N^6^-methyladenosine (m^6^A), 5-methylcytosine (m^5^C), N^1^-methyladenosine (m^1^A) and pseudouridine (ψ), among others, with m^6^A being the most abundant in eukaryotic cells, present in over 25% of human mRNAs and being enriched in long exons and near transcription start sites and stop codons (Gilbert et al., 2016; Li et al., 2016). Nearly every gene gives rise to both methylated and unmethylated mRNA (Molinie et al., 2016). In mammals, Mettl3, Mettl14, and other components form a methyltransferase complex that install m^6^A marks, while demethylases Fto and Alkbh5 remove m^6^A marks (Zhao et al., 2017). m^6^A modifications play diverse roles in stem cell function, development, and neuronal physiology by regulating mRNA processing, translation, and decay, through diverse m^6^A-binding proteins, for example, YT521-B homology domain family (Ythdf) proteins (Zhao et al., 2017).

The role of RNA modifications in CNS injury is largely unknown. A recent study linked m^6^A methylation to axon regeneration in conditioned DRG neurons (Weng et al., 2018). Peripheral axotomy elevated m^6^A-tagged mRNA levels in adult DRG neurons, and genome-wide profiles of m^6^A-tagged mRNA showed that the most enriched gene ontology terms involve translation and metabolism-related processes. A large number of RAGs (129 out of 304 examined) also exhibited significant m^6^A tagging in conditioned as compared to naïve DRG 24 hr after axotomy, including Atf3, Sox11, Gadd45a, and Tet3 (Weng et al., 2018) (**Table 2**). Interestingly, single-base mapping of m^6^A-tagging revealed dynamic changes at m^6^A-tagged sites, with some transcripts exhibiting a gain or loss of m^6^A sites across the 5’ UTR, coding regions, and 3’UTR, while other transcripts displaying region-specific changes. The transcripts with newly added m^6^A site were enriched for genes involved in axonal regulation, while transcripts encoding protein translation machinery components showed elevated m^6^A-tagged transcript levels, but not new m^6^A sites. Conditional deletion of Mettl14, a core unit of the mammalian m^6^A methyltransferase complex, resulted in reduced m^6^A levels in adult DRGs. Mettl14 and m^6^A-binding protein YTHDF1 were both required for peripheral axon regeneration in a sciatic nerve injury model (Weng et al., 2018). Finally, in the CNS axon regeneration model of optic nerve injury, PTEN-deletion-induced axon regeneration also required Mettl14 (Weng et al., 2018). Given the high prevalence of RNA modifications, a large amount of work is needed to map out epitranscriptomic changes and define their functional significance in regulating RAGs expression and regenerative capacity in different types of neurons.

## Regeneration-Associated Transcriptional Modules for Pro-Axon Growth Gene Programs

In addition to chromatin modifications, full scope activation of the regenerative gene program requires induction of key TFs, which help to recruit chromatin remodelers to specific genomic loci to modify DNA or histones. Indeed, it has been shown that co-expressed genes with coherent functions and similar transcriptional timing and levels may be co-regulated by transcriptional modules consisting of TFs and chromatin remodelers that collaborate with one another (Ram et al., 2011; Ernst et al., 2012). In this regard, TF binding to promoter elements of target genes helps to recruit chromatin modifiers to alter local chromatin structure, which in turn increases TF accessibility. Conversely, chromatin compaction precludes interaction between TFs and target loci. In the context of nerve injury and axon regeneration, specific transcriptional modules may be activated to regulate co-expressed genes for stress response, metabolic adaption, and axon regeneration, etc.

Our laboratory has elucidated one such transcriptional module consisting of HDAC1/p300 and Smad1 that regulates axon growth potential in DRG neurons through histone acetylation (**Figure 2A**). In embryonic DRG neurons, Smad1-mediated bone morphogenetic protein (BMP) signaling is active and is required for developmental axon growth (Finelli et al., 2013a). Turning down BMP/Smad1 signaling contributes to an age-associated decline of the axon growth potential (Zou et al., 2009; Parikh et al., 2011). Smad1 collaborates with p300 and HDAC1 for RAGs induction: promoter occupancy by Smad1 helps to recruit p300 and displaces HDAC1 at target loci, leading to histone acetylation, and this in turn increases Smad1 promoter binding (Finelli et al., 2013b). Reactivating developmentally regulated transcriptional modules may enable coordinated induction of a large network of pro-growth genes (Weng et al., 2016). Indeed, in the case of HDAC1/Smad1 signaling, both activating BMP/Smad1 signaling in adult DRG neurons by intrathecal delivery of adeno-associated virus 8 (AAV8)-BMP4 and elevating histone acetylation levels by HDAC1 inhibitor MS275 lead to enhanced axon growth potential and sensory axon regeneration in mouse models of SCI (Parikh et al., 2011; Finelli et al., 2013b). It remains to be seen whether combinatorial administration of AAV-BMP4 and MS275 can synergistically promote sensory axon regeneration in SCI models.

How Tet3 is targeted to specific regions of RAGs upon peripheral axotomy of DRG is also unsolved at this point, but may require collaboration with TFs as implicated in the genome-wide 5hmC mapping of conditioned DRG (Loh et al., 2017). Using bioinformatics analyses, we identified enriched TF binding motifs at DhMRs, including HIF (hypoxia-inducible factor), STAT, and IRF families, and these TFs may function as transcriptional coregulators to assist Tet3 for 5hmC modifications (**Figure 2C**). Notably, in DRG neurons, HIF1α has been shown to regulate multiple RAGs, and its activation is necessary and sufficient to promote peripheral axon regeneration in a sciatic nerve injury model (Cho et al., 2015). In *C. elegans*, *hif-1* mutants show reduced regeneration (Nix et al., 2014). Ongoing work is being conducted to define transcriptional modules composed of Tet3 and TFs such as HIF1α that regulate RAGs through 5mhC modifications. HIF can also form complexes with transcriptional coactivator CBP/p300 (Lisy and Peet, 2008) or chromatin-remodeling complexes (Dekanty et al., 2010). Indeed, HIF1α is required for injury-induced histone H3 acetylation (Cho et al., 2015).

Consistent with the notion of regeneration-associated transcriptional modules, system-level transcriptional network analysis revealed tight co-expression of transcriptional regulators after peripheral axotomy of DRG neurons, which in turn coordinate activation of the regenerative gene program (Chandran et al., 2016). Additionally, transcriptional profiling across early stages of PNS regeneration revealed a clear cascade of target genes activation after axonal injury, starting with a wave of chromatin modification followed by activation of RAGs (Li et al., 2015).

The importance of collaboration between TFs and chromatin remodelers for RAGs induction has been further supported by the finding that epigenetic constraints might actually limit the full efficacy of pro-regenerative TFs (RAG-TFs) even in the setting of overexpression. In a study of ascending sensory axon regeneration after SCI, AAV5 was used to deliver ATF3 alone or in combination with c-JUN, STAT3C (a dominant active mutant form of STAT3) and Smad1-EVE (a constitutively active phospho-mimetic form of Smad1) into lumbar L4 and L5 adult rat DRG (Fagoe et al., 2015). These four RAG-TFs each individually contributes to regenerative axon growth of DRG neurons after peripheral axotomy. It was found that central axon regeneration of DRG neurons was enhanced with ATF3 expression, but no synergistic effect was observed when expressing all the four TFs as compared to ATF3 alone. Thus, overexpressing multiple RAG-TFs was still insufficient for full scope activation of the regeneration program. In another study using dorsal hemisection SCI model, descending corticospinal tract (CST) regeneration was compared with overexpression of SOX11, VP16-KLF7 (addition of a VP16 activation domain), VP16-STAT3, or c-JUN (Venkatesh et al., 2018). While SOX11 and KLF7 stimulated axon regeneration from CST motoneurons, STAT3 and c-JUN did not. It was revealed that the chromatin accessibility of the predicted STAT3 and JUN target genes was lower than that of SOX11 and KLF7 target genes. Thus, to be effective, RAG-TFs either need to have their binding sites sufficiently accessible, or they must be capable of recruiting chromatin regulators to modify chromatin accessibility. Venkatesh et al. (2018) further proposed that a collaboration between pioneer TFs and RAG-TFs might be needed to achieve full efficacy. Pioneer TFs are capable of binding to compacted chromatin and initiate a cascade of molecular events to reshape the chromatin landscape (Zaret and Carroll, 2011; Zaret, 2018). Computational algorithms based on genome-wide chromatin accessibility datasets have recently been developed for chromatin opening index/pioneer index of TFs (Sherwood et al., 2014; Lamparter et al., 2017). Venkatesh et al. went on to use the pipeline to predict pioneer factors that could potentially relieve chromatin constraints for STAT3 or c-JUN target genes. In future studies, it will be exciting to test experimentally whether the rational design of combinatorial activation of TFs and epigenetic factors or pioneering TFs is capable of reversing the seemingly unamendable chromatin constrains of RAGs in adult CNS neurons, leading to synergistic regenerative effect.

## Developmental Decline of Axon Growth Potential and Associated Progressive Change of Chromatin Landscape

The regenerative state in adult neurons frequently involves reactivating the developmental program. During neurodevelopment, as embryonic neurons establish axon wiring and synapses mature, a developmental decline in intrinsic axon growth potential occurs (Liu and Snider, 2001; Moore et al., 2009; Parikh et al., 2011; He and Jin, 2016; Tedeschi and Bradke, 2017). During this process, pro-axon growth genes are turned off and growth-inhibitory genes are upregulated. It is now clear that this developmental transition is accompanied by progressive changes of the chromatin landscape to ensure stable gene expression after neuronal maturation (Venkatesh et al., 2018). Unfortunately this restrictive chromatin status becomes an epigenetic barrier for reactivating the pro-growth gene networks, thus finding ways to unlock the epigenetic blockade promises to rejuvenate the growth competence in adult neurons (Wong and Zou, 2014).

Early insights into the epigenetic barriers for regeneration in mature neurons came from studies tracking the changes in histone acetylation levels across developmental stages. In cortical neurons and CGNs, global levels of H3K9ac and H3K14ac decline during development, while in RGCs, H3K18ac level declines as neurons mature (Gaub et al., 2010; Gaub et al., 2011) (**Table 1**). Consistently, chromatin accessibility at promoter regions of several RAGs displays developmental decline during cortical maturation, as evidenced by depletion of H3K4me3, a marker for euchromatin, and enrichment of H3K27me3, a marker for heterochromatin (Venkatesh et al., 2016) (**Table 1**). The recent development of the ATAC-seq method (assay for transposase-accessible chromatin using sequencing) allowed direct measurement of chromatin accessibility (Buenrostro et al., 2013), and demonstrated a genome-wide shift in chromatin accessibility in adult mouse cortex as compared to neonatal cortex, with inaccessible regions being highly enriched for gene ontology terms related to axon growth and guidance, cell-cell adhesion and GTPase signaling (Venkatesh et al., 2018). Hence, from a developmental perspective, once axon wiring is completed, chromatin remodeling serves as a safeguard mechanism to restrict accessibility of pro-growth genes. Identifying the underlying epigenetic mechanisms may provide an avenue to promote regeneration through reactivation of developmental programs.

The epigenetic factors that regulate the developmental changes in chromatin landscape are still being investigated. p300 has been shown to be developmentally regulated in RGCs and its expression remains repressed after optic nerve injury (Gaub et al., 2011). By comparison, the developmentally regulated TFs that control intrinsic axon growth capacity comprise an ever expanding list, including ATF3, c-JUN, CREB, Krüppel-like family of TFs (KLFs), Smad1, Sox11, and STAT3 (reviewed in Weng et al., 2016). Many of these TFs also regulate intrinsic growth competence in adult CNS neurons, including Smad1 (Finelli et al., 2013a), c-JUN and KLFs. KLF-based manipulations improved axon regeneration of RGCs after optic nerve injury and CST motor neurons after SCI (Moore et al., 2009). A comprehensive comparison has been conducted across four transcriptomic datasets to identify converging intrinsic axon growth modulators, which identified c-JUN as a common TF to all four datasets, where as ATF3 and members of the KLF and Smad families were found in three datasets and Yy1, Fos, Egr1, Isl2, and members of the STAT, Sox, and Gata families were shared by two datasets (Blackmore, 2012). Whether these developmentally regulated TFs collaborate with epigenetic factors to form pro-growth transcriptional modules remains to be investigated, particularly in the context of CNS neurons.

## Signaling Pathways in Organizing Transcriptional Hierarchy of Regenerative Gene Program

Many signaling pathways that regulate axon growth potential have been identified by functional genetic screens, proteomic approaches, and system-level analyses, including PTEN/mTOR, JAK/STAT, and DLK/JNK pathways (Liu et al., 2010; Belin et al., 2015; Chandran et al., 2016) (also see review in Lu et al., 2014). A recent *in vivo* screen searched for modulators of axon sprouting after SCI by comparing the transcriptomes of intact CST motor neurons that remained quiescent versus those that exhibited injury-induced sprouting, which revealed a set of pro-axon growth pathways including HIPPO signaling, mTOR signaling, and the 3-phosphoinositide degradation pathway (Fink et al., 2017). Signaling pathways might play an active role in organizing transcriptional hierarchy to reestablish axon growth competence (Weng et al., 2016). For instance, ERK-mediated retrograde signaling is required for PCAF-mediated histone acetylation at RAG promoters (Puttagunta et al., 2014) (**Figure 2B**). One important area of research is to examine whether signaling pathways act in concert with chromatin remodelers and RAG-TFs. A recent review discussed the striking convergence of disparate regeneration-associated biological processes on chromatin accessibility (Danzi et al., 2018). For instance, PTEN deletion promotes axon regeneration, which has been attributed to enhanced mTOR signaling (Park et al., 2008). Interestingly, PTEN can also directly interact with histone H1, and PTEN deletion prevented binding of H1 to DNA and increased H4K16ac, thereby promoting chromatin accessibility (Chen et al., 2014). PTEN also antagonizes insertion of histone variant H3.3, which is associated with transcriptional activity and chromatin accessibility (Benitez et al., 2017). Furthermore, neuronal activity-induced axon growth competence may also involve increased chromatin accessibility, e.g. at AP-1 motif (Su et al., 2017).

It is important to bear in mind that pro-growth epigenetic factors and signaling pathways may not only induce growth-promoting genes, but also suppress growth-restrictive genes. For instance, both PTEN and KLF4 suppress neurite outgrowth in RGCs (Park et al., 2008; Moore et al., 2009). Interestingly, inhibition of S6 kinase I (S6K1), a prominent mTOR target, in fact promotes neurite outgrowth of primary hippocampal neurons, as well as CST regeneration in a dorsal hemisection SCI model *in vivo*, and this occurs through activation of PI3K/mTOR signaling by way of a negative feedback mechanism (Al-Ali et al., 2017).

## Glial Epigenetics in Tissue Repair After CNS Injury

The glial cell compartment, which make up to ∼90% of cells in the CNS, has received little attention in regards to epigenetics in shaping their injury responses. CNS injury triggers a complex, multiphasic glial responses, with both beneficial and detrimental effects (reviewed in Staszewski and Prinz, 2014). Specific patterns of chromatin modifications are initiated in different glial cell types as a result of complex interactions between the type of injury, injury microenvironment, intrinsic factors, and the epigenome unique to each cell types.

## Epigenetic Regulation of Neuroinflammation

**Figure 3:**
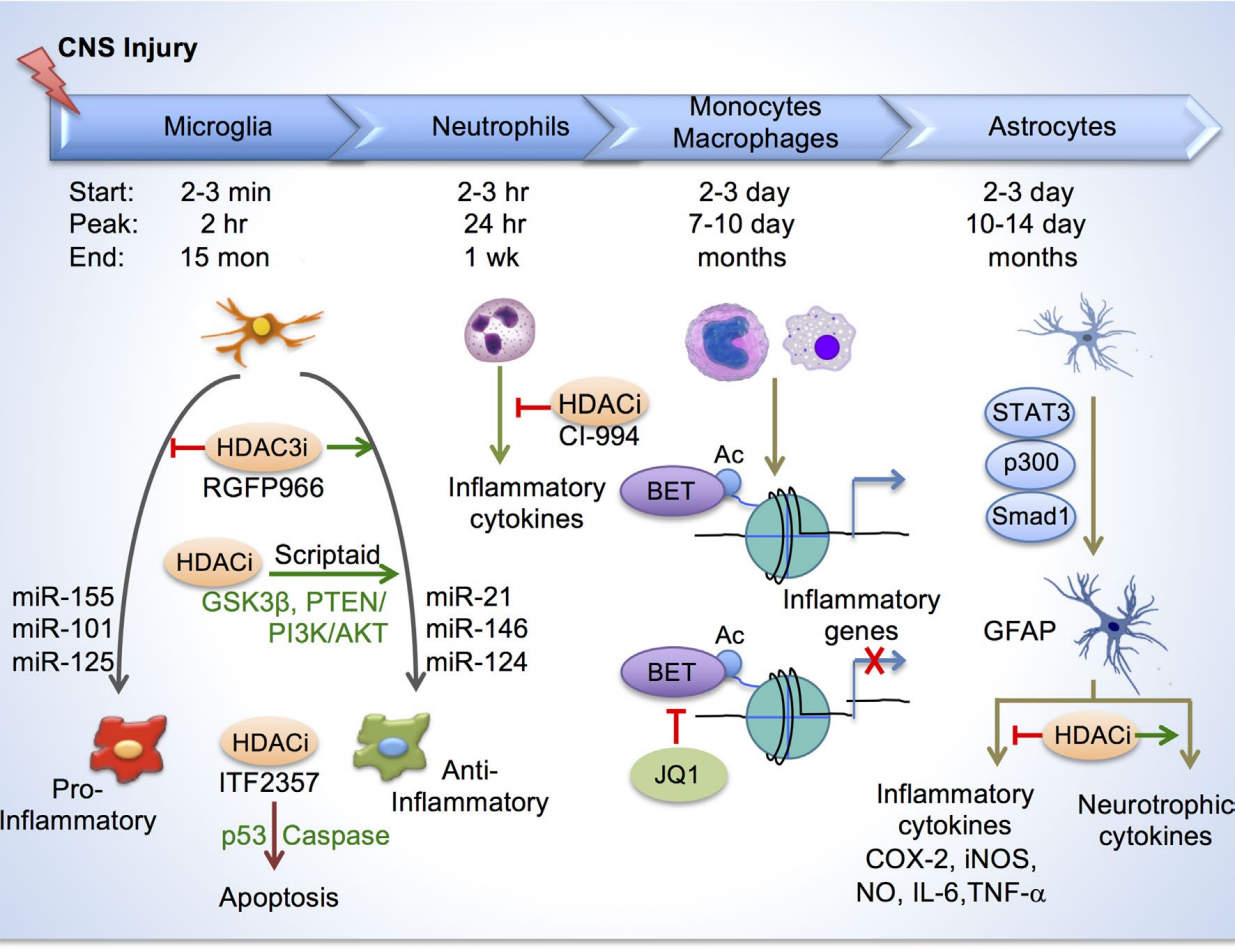
Histone acetylation in regulating glial response after CNS injury. Top, depiction of timelines of activation of different immune cells and astroglia at the injury site after CNS injury. Bottom, in microglia/macrophages, HDAC inhibition reduces inflammation by enhancing anti-inflammatory/pro-repair phenotype and by inducing apoptosis through p53 and caspase. Different micro-RNAs also regulate inflammatory phenotypes of microglia/macrophage. HDAC inhibition by CI-994 suppresses neutrophil accumulation and reduces inflammatory cytokine expression. BETs are epigenetic readers of acetylated histones and promote transcription of inflammatory genes. BET inhibitor JQ1 reduced inflammatory cytokine expression and leukocyte recruitment to the injury site. In astrocytes, transcriptional module consisting of STAT3, p300 and Smad1 induces GFAP expression. HDAC inhibition reduces secretion of inflammatory cytokines, and increases neurotrophic cytokines from reactive astrocytes.

Inflammatory responses in the CNS are mediated by resident microglia, blood-borne macrophages and many other immune cell types, such as neutrophils, T cells, and B cells, as well as astrocytes (**Figure 3**). Following injury, resident microglia are activated within minutes, and blood-borne monocytes infiltrate the injury site to differentiate into phagocytic macrophages, which share similar morphology and cell-surface markers, and together they constitute the innate immunity (Beck et al., 2010). In response to injury signals, microglia/macrophages can adopt extensive functional heterogeneity with multitude of roles, including phagocytosis, debris clearing, release of inflammatory mediators, promoting angiogenesis, and supporting neuronal survival (reviewed in Garden, 2013). Each of these tasks engages unique gene networks regulated by distinct epigenetic mechanisms, which may serve as useful therapeutic targets for immunomodulation after CNS injury.

Inflammatory responses compose a spectrum of macrophage activation phenotypes, ranging from a pro-inflammatory, classical activation state (also known as M1 polarization) to an anti-inflammatory, alternative activation state (M2 polarization)(Martinez and Gordon, 2014). M1 polarization is mediated by type 1 T-helper (Th1) cytokines such as interferon-gamma (IFNγ) and Toll-like receptor (TLR) agonists such as lipopolysaccharides (LPS), and is associated with cytotoxic tissue injury. In contrast, M2 polarization is induced by type 2 T-helper (Th2) cytokines such as interleukin-4 (IL-4) and interleukin-13 (IL-13), and is thought to promote tissue repair (**Figure 3**). While both differentiation states have been reported in CNS injury (Kigerl et al., 2009), the epigenetic mechanisms regulating these activation states and the degree of plasticity between different states are still being investigated.

Histone acetylation plays a critical role in shaping the inflammatory responses of microglia/macrophages. Broad spectrum HDACi have been used to demonstrate the overall impact on improving functional recovery in various CNS injury paradigms, including stroke, experimental autoimmune encephalitis (EAE), SCI, and traumatic brain injury (TBI) (reviewed in Garden, 2013). After CNS injury, H3ac levels decline, thus HDACi-mediated neuroprotection is thought to occur by restoring adequate histone acetylation levels; however, because HDACi affects a variety of cells, and both histone and non-histone targets, including key inflammatory regulators, the underlying mechanisms are likely multifactorial.

In a recent study, we elucidated a role of HDAC3 for epigenetic regulation of inflammatory responses in microglia/macrophages in a contusion SCI model (Kuboyama et al., 2017). We found that HDAC3, but not other class I HDACs (HDAC1, 2, 8), was robustly upregulated in activated microglia/macrophages after SCI. Administration of RGFP966, a small-molecule inhibitor that specifically targets HDAC3, resulted in global suppression of inflammatory cytokines at the injury site, and improved neuroprotection and functional recovery after SCI (**Figure 3**). Mechanistically, in primary microglia, HDAC3 activity contributes to histone deacetylation and the inflammatory responses to LPS, a classic inflammatory stimulus (Kuboyama et al., 2017). Consistently, in non-CNS injuries, HDAC3 is shown to function as an epigenetic brake in macrophage alternative activation (Mullican et al., 2011). Furthermore, HDAC3 is required for upregulation of an inflammation-associated gene program in macrophages, as macrophages lacking HDAC3 became less responsive to IFNγ stimulation, but hypersensitive to IL-4 stimulation (Chen et al., 2012). Future directions worth pursuing include conditional ablation of HDAC3 in microglia/macrophages in CNS injury models, and identifying specific genomic loci that are under direct regulation of HDAC3.

In TBI, treatment with HDACi Scriptaid also shifted microglia/macrophage towards a protective M2 phenotype and mitigated inflammation through upregulation of GSK3β, which can modulate microglial functions *via* the PTEN/PI3K/Akt signaling pathway (Wang et al., 2015). In another study, HDACi treatment after TBI attenuated inflammation by promoting apoptosis of activated microglia/macrophages through a mechanism involving p53 upregulation and caspase activation (Shein et al., 2009) (**Figure 3**).

Recently, another class I HDACi, CI-994, has been shown to promote motor functional recovery following SCI, and this was attributed to a neuroprotective effect since no increased sprouting of CST fibers were observed (Zhang et al., 2018b). In a SCI model, CI-994 treatment resulted in increased H3ac levels in neutrophils and microglia/macrophages at the injury site, and suppressed neutrophil accumulation, reduced inflammatory cytokine expression and decreased neuronal loss as early as three days following injury. Bromodomain and Extra-Terminal Domain-containing proteins (BETs; Brd2, Brd3, Brd4, BrdT) are epigenetic readers that bind acetylated histones and promote transcription of proinflammatory genes. Administration of BET inhibitor JQ1 in SCI model resulted in reduced proinflammatory cytokine expression and leukocyte recruitment to the injury site by three days post injury; however, these changes did not appear to lead to improved functional recovery or smaller lesion size (Rudman et al., 2018).

Other epigenetic modalities are less understood in mediating the innate immune response in CNS injury. In microglia-like BV-2 cells, DNA methylation can influence expression of Alzheimer’s Disease (AD) associated genes by way of hypomethylation of gene promoters (Lin et al., 2009). TBI reportedly induces hypomethylation mainly in ∼10–20% of activated microglia/macrophages in injury area as early as day one (Zhang et al., 2007), but the functional significance is unclear. Micro-RNA expression profiles in microglia are distinct from that of neurons and other glial cells (Jovičić et al., 2013). miRNAs can regulate differentiation and activation of cells of the immune system (Ponomarev et al., 2013). Pro-inflammatory miRNAs included miR-155, miR-101 and miR-125. On the other hand, MiR-21 and miR-146 were also induced in macrophages by the classic proinflammatory stimuli LPS, but they promoted a resolution of inflammation (reviewed in Ponomarev et al., 2013). Brain-specific miR-124 is expressed in homeostatic microglia but absent in peripheral macrophages. miR-124 promotes microglia quiescence; and consistently, it is downregulated in activated microglia in EAE or after treatment with proinflammatory cytokines such as IFNγ and GM-CSF. Treatment of mice with miR-124 inhibits EAE and reduces CNS inflammation (Ponomarev et al., 2011). In the cancer field, advances have been made in understanding epigenetic regulation of T cell dysfunction and therapeutic programmability. For instance, T cells in tumors are dysfunctional due to immune checkpoint blockade. T cells have been shown to differentiate through two distinct chromatin dysfunctional states–a plastic and a fixed state–as a result of heritable, epigenetically imprinted mechanisms (Philip et al., 2017). The functions of these epigenetic mechanisms in influencing immune responses and neural repair in CNS injury awaits future investigation.

Axonal injury also elicits a glial response located near the soma of injured neurons or surround axons. For instance, peripheral but not central axotomy leads to significant macrophage accumulation in the DRG, which increases regenerative capacity of DRG neurons, and this occurs through chemokine CCL2 (Niemi et al., 2013; Niemi et al., 2016).

## Epigenetics of Reactive Astrocytosis for Neural Repair

Astrocytes are also a key glial component that contributes to inflammatory responses (Gao et al., 2013; Goldmann et al., 2016). HDAC activity is increased in astrocytes under inflammatory conditions, and HDAC inhibition reduces glial inflammatory responses in both microglia and astrocytes (Faraco et al., 2009; Suh et al., 2010). During differentiation of neural progenitors into astrocytes, upregulation of GFAP, an astrocyte marker, requires recruitment of transcriptional module comprised of STAT3, p300 and Smad1 to its promoter (Nakashima et al., 1999). During reactive astrocytosis, GFAP is upregulated; and HDAC inhibition reduced GFAP expression in primary human astrocytes (Kanski et al., 2014), but did not reduce astrocyte activation (Xuan et al., 2012). Reactive astrocytes upregulate cyclooxygenase (COX)-2, iNOS, NO, IL-6 and TNF-α, which can be attenuated by HDAC inhibition in different CNS injury scenarios (reviewed in York et al., 2013). Reactive astrocytes also increase production of glycosaminoglycans, such as chondrointin sulfate proteoglygan (CSPG), some of which can act as HAT inhibitors, thereby decreasing acetylation levels in neighboring cells (Buczek-Thomas et al., 2008). In co-cultures, HDAC inhibition increases secretion of neurotrophic cytokines by astrocytes, resulting in neuroprotection of dopaminergic neurons (Chen et al., 2006). Taken together, epigenetic modulation may be a powerful tool to influence astroglial responses, thereby promoting neuroprotection and neuronal repair after CNS injuries (York et al., 2013).

## Caveats and Future Directions

One limitation of current approaches is the use of whole neural tissue for epigenomic studies, which does not distinguish cell-type specific epigenetic changes. For instance, whole DRG consists of sensory neurons and a larger population of glial cells, including macrophages and Schwann cells. There are also many different neuronal subtypes in DRG. Indeed, single cell RNA-Seq revealed heterogeneous transcriptional responses of DRG neuronal subtypes after nerve injury (Hu et al., 2016). Another study found that in DRG, DNMT1 is expressed in both glia and neurons, DNMT3a is preferentially expressed in glia, while DNMT3b is preferentially expressed in neurons but absent in glia; and all are upregulated in a model of nerve injury (Pollema-Mays et al., 2014). Hence, different epigenetic factors are involved in injury responses and transcriptional changes in different cell types in neural tissues.

New epigenetic mechanisms continue to come to light, and their roles in CNS injury are poorly understood. For instance, non-canonical histone variants such as H2A.Z and H3.3 can influence chromatin architecture and genomic function (Henikoff and Smith, 2015). Nucleosome repositioning or sliding along DNA as mediated by adenosine triphosphate (ATP)-dependent chromatin-remodeling enzymes can control DNA accessibility by transcriptional machinery (Mueller-Planitz et al., 2013). Higher-order chromatin remodeling, such as DNA looping, chromatin boundary elements, functional nuclear domains also affect chromatin organization (Mercer and Mattick, 2013). Epigenetic mechanisms also regulate mitochondria gene expression (Shock et al., 2011), which may be important for metabolic adaption during axon regeneration and glial activation.

Individual RAGs may employ multiple epigenetic mechanisms for their transcriptional regulation in a cell-type specific manner, and different epigenetic mechanisms can interact with one another (**Table 2**). For instance, DNA demethylation may coordinate with histone acetylation or other epigenetic mechanism to promote axon regeneration (Trakhtenberg and Goldberg, 2012). CpG methylation can influence histone methylation patterns. For example, binding of MBD proteins to the methylated CpG can recruit histone modifiers to maintain transcriptional repression (Weng et al., 2016). Conversely, H3K4 methyltransferase can be recruited to non-methylated CpG islands to enhance H3K4me3 levels and activate transcription. Epigenetic and epitranscriptomic mechanisms may also interact, e.g. Tet3 and Gadd45a transcripts are both targets of m^6^A mRNA modifications (Weng et al., 2018).

New methods, such as a CRISPR-dCas9-based system, now allow activation of target genes by locally rewriting gene-specific epigenetic codes, thus circumventing off-target effects or avoiding pharmacological approaches that ubiquitously modify histone codes (Liao et al., 2017; Xie et al., 2018). Combinatorial epigenetic strategies are worth exploring, with the aim to achieve simultaneous enhancement of neuronal intrinsic axon growth potential and modulation of glial inflammatory responses. It is important to first delineate cell type-specific roles of individual member of the epigenetic factors in CNS injury. For instance, HDAC1 and HDAC5 regulate RAG expression and axon growth potential in DRG neurons (Cho et al., 2013; Finelli et al., 2013b), while HDAC3 controls inflammatory gene programs in activated microglia (Kuboyama et al., 2017). It is worth noting that HDAC3 also functions as a key regulator of apoptotic gene silencing in RGCs after optic nerve injury (Pelzel et al., 2010). HDAC3 was shown to translocate to the nuclei of dying RGCs as an early response to axonal injury, and this is associated with widespread H4 deacetylation and transcriptional dysregulation in dying RGCs. Conditional knockout of *Hdac3* or blocking HDAC3 activity using RGFP966 in a dose dependent manner prolonged RGC survival through protection against nuclear atrophy and apoptosis (Schmitt et al., 2014; Schmitt et al., 2018).

## Conclusion

Here, we summarized recent progress in understanding the roles of chromatin accessibility, microRNAs, and epitranscriptomics in fine-tuning transcriptional changes in neurons and glia in response to axonal injury. These advances provide a framework for defining how adaptive and maladaptive epigenetic changes impact intrinsic axon regeneration competence and glial activation in the context of neural repair. Given the broad scope and complexity of epigenetic mechanisms, more work is needed before epigenetic strategies can be combined with other treatment modalities to promote functional recovery after CNS injury. 

## Author Contributions

All authors contributed to manuscript preparation. SW, DH, and XZ performed literature search and prepared the diagrams and tables, and HZ, SW, and DH wrote the review.

## Funding

This work was supported by grants from the National Institutes of Health (R01/R56 NS073596), Craig H Neilsen Foundation (#476516), and New York State Spinal Cord Injury Research Board (DOH01-C32242-GG, -C33268GG) to HZ, and postdoctoral fellowship grants from New York State Spinal Cord Injury Research Board (DOH01-C32634GG) to SW, and from the Chinese Scholarship Council to XZ.

## Conflict of Interest Statement

The authors declare that the research was conducted in the absence of any commercial or financial relationships that could be construed as a potential conflict of interest.
